# Organic Acids Mixture as a Dietary Additive for Pigs—A Review

**DOI:** 10.3390/ani10060952

**Published:** 2020-05-30

**Authors:** Dinh Hai Nguyen, Woo Jeong Seok, In Ho Kim

**Affiliations:** Department of Animal Resource and Science, Dankook University, Cheonan-si, Chungnam 31116, Korea; nguyenhaibg90@gmail.com (D.H.N.); sukooj@naver.com (W.J.S.)

**Keywords:** organic acids, pigs, microbial interactions, gastrointestinal tract

## Abstract

**Simple Summary:**

Many countries have enforced a ban on the use of all antibiotics in animal feed as growth promoters due to public health concerns about the emergence of antimicrobial resistance. There are a variety of candidates for the replacement of antibiotic growth promoters like probiotic, prebiotics, herbs, or essential oil. Organic acids have been suggested as alternatives to replace antibiotics in the diets of animals. It is reported that the lower the pKa of the OAs, the greater its effect on the reduction of stomach pH and the lower its antimicrobial effect in the more distal portions during its transit through the GIT which eventually will have better effect on animal’s health as well as their performance. Herein, we focus on the use of organic acids’ mixture as feed additives in the diet of swine in term of their immune system, gut health, nutrient digestibility, and growth performance as well as gas emission.

**Abstract:**

Due to the increasing safety concerns about the risk of spreading antibiotic resistance in the environment, and the presence of chemical residues in animal products, using organic acids (OAs) to replace antibiotic in the diet of farm animals has increased considerably in recent years. It has been suggested that OAs could attribute to diverse elements such as antimicrobial activity, decreasing the pH of digesta particularly in the gastrointestinal tract (GIT), slowing feed transit in the GIT to maximize feed digestion and nutrient absorption, inducing enzyme secretion and activity in the small intestine, and providing nutrients to intestinal tissue. It has been reported that OAs mixture might be more effective than individual OAs due to the synergistic effects of different pKa values and have a broad-spectrum activity. In conclusion, this review showed that an OA mixture, which can improve nutrient digestibility and growth performance, modulate intestinal bacterial populations and improve gut health, as well as decreasing gas emission, can be used as alternative to antibiotic growth promoters. However, the results of OA mixtures are not always consistent, and the response to dietary OAs could be affected by the type of OAs, dosage, feed formula, and the age of animals. In this review, we will give an overview of the current use of OAs mixture in swine feed.

## 1. Introduction

Intestinal microorganisms influence their hosts owing to their various physiological functions [1]. When gut health is compromised, digestion and nutrient absorption are affected which, in turn, can have a detrimental effect on feed conversion leading to economic loss and a greater susceptibility to disease [2]. Natural intestinal microflora adhere to the intestinal mucosa and inhibit colonization by pathogens due to the competition for nutrients and binding sites, bacteriocin production, lactobacilli fermentations, and short-chain fatty acids (SCFA) production [2]. Before 2006, antibiotics were widely used as health strategies to inhibit enteric infections and the occurrence of pathogens able to adhere to intestinal mucosa [3]. Their effectiveness in improving growth rate, feed efficiency, and decreasing mortality is well documented [4]. However, growth promoting antibiotics have been banned for 15 years in animal production due to the concern of antibiotic resistance in pathogens.

During past several decades, the world is becoming increasingly concerned about drug residues in meat products [2]. In addition, it has been suggested that the continuous use of antibiotics may contribute to a reservoir of drug-resistant bacteria that may be capable of transferring their resistance to pathogenic bacteria in both animals and humans. To maintain animal health and performance, many researchers have suggested that the use of organic acids (OAs), organic minerals, bacteriophages, eubiotics, phytobiotics, probiotics, and prebiotics as potential alternatives when antibiotic growth promoters are withdrawn from animal feed. Among these alternatives, dietary OAs have been broadly applied worldwide because of their antimicrobial activity, which can induce a pH reduction in the gastrointestinal tract (GIT) [5], play a role against pathogenic bacteria, and eventually enhance nutrient utilization, and growth performance of animals [6]. Partanen and Mroz (1999) reported that the inclusion of OAs in the diet can enhance growth performance and modulate intestinal microbiota in pigs [7]. Lactic acid has been reported to reduce gastric pH and delay the multiplication of an enterotoxigenic *Escherichia coli (E. coli)* [8] and to be more effective than other OAs in improving the growth performance of pig [9]. Other studies have demonstrated that, under normal physiological conditions, dietary inclusion of OAs was beneficial to swine and improved growth performance and reduced the post-weanling diarrhea in piglets challenged with *E. coli* K88 [10], reduced fecal pH and increased serum lymphocyte concentrations, and improved growth performance of growing pigs [11]

The most commonly used organic acids are short chain fatty acids, e.g., formic acid, propionic acid, butyric acid, acetic acid, citric acid, and malic acid, which is a dicarboxylic acid. These all are usually weak organic acids, which, when dissolved in water, change into their hydrogen and hydroxyl ion, respectively. The working and mode of action of these acids are dependent upon their pH and Pka value. Some of the most commonly OAs used in animal nutrition are listed in Table 1 [12,13]. In addition, OAs have some additional effects that go far beyond antibiotics such as lowering digesta pH and increasing the levels of pancreatic secretion [10]. It has been reported that an individual acid has its own range of microbial activity in terms of physiology, pH range, and membrane structure. Generally, the mixture or combination of acids shows different pKa values and have a broad-spectrum of activity, thereby maintaining optimum pH through the intestinal tract [14].

The purpose of this review is to assess the response of pig to OAs mixture of the previous reports using as demonstrated in terms of growth rate, feed intake (FI), and feed conversion ratio (FCR). In addition, reasons for the variable responses to and possible modes of action of OAs will be discussed.

## 2. Modes of Action of Organic Acids

Like antibiotics, OAs have an antimicrobial activity, which has a clear and significant benefit on gut health and development, and eventually leads to a positive effect on animal health and their productivity. The positive effects of OAs may be attributed to various factors, including (1) antimicrobial activity of non-dissociated OAs; (2) lowering the pH of digesta particularly in the stomach, aiding protein digestion; (3) decreasing the emptying rate of stomach; (4) stimulating excretion and activity of enzyme (pancreatic) in the small intestine; and (5) supplying nutrients to intestinal tissue; therefore, improving mucosal integrity and function [15].

### 2.1. Effect of Organic Acids on Antimicrobial Activity and Lowering pH

Theobald (2015) reported that the efficacy of an acid in inhibiting microbes is dependent on its pKa value, which is the pH at which 50% in its dissociated and non-dissociated form respectively [16]. Non-dissociated OAs can freely penetrate the semipermeable membrane of the microorganisms into the cell cytoplasm and change their metabolism [17]. This means that the antimicrobial effect of OAs is greater under acidic conditions, like in the stomach, and lesser at neutral pH, like in the intestine. To achieve the optimal growth, most species of bacterial require specific pH and they are unable to grow under extreme acidic conditions (pH < 4.5). OAs can influence their antimicrobial activity against bacteria by directly lowering the pH of the environment through the release of hydrogen ions and thus preventing or inhibiting the proliferation of acid-sensitive bacteria. It is well known to use weak OAs like lactic, sorbic, benzoic, citric, and acetic acids by lowering the pH of foods or beverages to impede microbial growth [18].

The pKa values of OAs and the pH conditions of the GIT will influence the pH reduction degree in diets and digesta [5]. The higher pKa value of OAs, the more effective preservatives for feed due to the weaker acids with a higher proportion of their non-dissociated form, and it can defend feed from being infected by fungi and microbes [16]. Therefore, the lower the pKa of the OAs (the higher proportion of dissociated form), the greater its effect on the reduction of stomach pH and the lower its antimicrobial effect in the more distal portions during its transit through the GIT. Thus, it is clear that each acid has a microbial activity spectrum involved to a specific pH range, membrane structure and physiology in the cell of the microbiota species, and acids mixture represent a range of pKa value and are used owing to the wider activity spectrum [19].

These researches explained that the main basic principle of the mode of action of OAs on bacterial is that non-dissociated OAs can penetrate the bacteria cell wall and break down the normal physiology of certain types of bacteria that we call “pH-sensitive” meaning that they cannot tolerate wide internal and external pH gradient.

### 2.2. Effect of Organic Acids on Nutrient Digestibility

Nutrient digestibility might be increased when the pH in the upper region of the digestive tract decreases. OAs, normally used as an acidifier in livestock feeds, have been considered to be promising antibiotics alternatives for enhancing nutrient digestibility. OAs improve protein digestibility by lowering the chyme pH. It is reported that the administration of OAs improves pepsin activity and microbial phytase activity, and, because of the lower pH [20,21], induce pancreatic excretion and increase trypsinogen, chymotrypsinogen A, chymotrypsinogen B, procarboxy peptidase A, and procarboxypeptidase B concentration, which all result in greater protein digestion [22]. According to Van Der Sluis 2002 [21], OA supplementation has a positive effect on digestion by slowing feed transit in the GIT, leading to better absorption of the essential nutrients. 

Moreover, by reducing the pH of the diet, OAs may improve the utilization of minerals [23]. It is also demonstrated that citric acid can help to release minerals bound to the phytate molecules, which in turn increases the utilization of Ca, P, and Zn [24]. Partanen et al. documented that formic acid tends to decrease bacterial nitrogen in different parts of the small intestine in pigs and improve apparent ileal digestibility of protein sources, certain essential amino acids, lipids, calcium, and phosphorus [25].

Besides, lower microbial proliferation in the GIT caused by the antimicrobial activity also plays a key role, which reduces the competition between the microflora and the host. This reduction in competition is one of the mechanisms responsible for improved digestibility [26].

### 2.3. Effect of Organic Acids on Pathogenic Bacteria

In general, the presence of bacteria in the GIT also results in the competition for nutrients between the bacterial population and the host animal. In addition, bacteria secrete toxic compounds (i.e., toxic amino acid catabolites) and reduce the ability to digest fat, induce rapid turnover of absorptive epithelial cells, require an increased rate of mucus secretion by intestinal goblet cells, and induce inflammatory responses and immune system development. All of these effects result in compromising growth rate, and the study has reported that the net energy can be consumed by microflora by up to 6% in pig diets [13]. Thus, it is important to handle harmful bacteria. Davidson et al. (2001) noted that the bacteria cell wall was susceptible to penetration, disrupting normal cellular functions by OAs, including replication and protein synthesis of bacteria [27]. The proposed sequential mechanisms of bactericidal action are followed as (1) the acid form of OAs (protonated form) can penetrate through the cell wall of bacterial and (2) penetrated OAs within bacterial cells dissociate into the conjugated base form (non-protonated form) with a concomitant reduction in cellular pH, creating a stressful environment due to pH reduction that leads to cellular dysfunctions, and thus preventing bacterial growth, especially pH-sensitive bacteria species [28]. It is reported that dietary citric acid inclusion or blend of OAs (including citric, formic, fumaric, and lactic acids) supplementation have benefit effects on microbial populations in the GIT [29,30].

Van Dam et al. (2006) suggested that OAs, such as formic, benzoic, lactic, and propionic acids, have been used to protect feed ingredient from fungi and microbes during storage time [31]. Some OAs have been proven to have strong antibacterial effects to prevent important foodborne pathogens such as *E. coli* and *Salmonella* spp. [32] and are currently used to handle bacterial pathogens in terrestrial animal feeds [33]. In addition, OA mixtures were found to have better effectiveness than Enramycin, an antibiotic, in reducing *E. coli* and Salmonella counts in intestine [34].

## 3. Protected Organic Acids

It was reported that the effectiveness of unprotected OAs may be limited because of prompt metabolism and absorption in the duodenum part that can affect to the ability of modulating intestinal flora due to lowering the amount of OAs that reaches the lower gut [35,36]. Improving the retention time of the nutrients and active compounds in the food and drugs microencapsulation technology, as one of the protection methods, has been used in the food and pharmaceutical industries for several decades [37]. Microencapsulation technology was first commercially applied in 1954. Shahidi and Han (1993) sketched out the reasons why the food industry applies microencapsulation: (1) reducing the reactivity of the core with external environment such as light, oxygen, and water; (2) preventing lumping, promoting easy mixing of the core material; (3) controlling the release of the core material to achieve the proper delay for the right stimulus; (4) concealing the core flavor; and (5) diluting the core material when it is used in only small quantities but still achieve uniform distribution [38]. Recently, it has been noted transport of SCFA further down the GIT by microencapsulation in a lipid shell, the protective lipid matrix used for microencapsulation allows OAs to have an effect all along the gastrointestinal tract, as they are slowly released during digestion [39].

According to Gheisari et al. (2007), dietary 0.2% protected OAs administration increased the populations of beneficial microflora (*Lactobacillus* spp.) and reduce harmful bacteria counts (Clostridium perfringens, *E. coli* and *Salmonella* spp.) in animal gut contents allowing OAs to act all along the GIT [40]. Smulikowska et al. (2009) noted that supplementation of protected OA in animal diet improved N retention compared to control diet [41]. Previous studies also indicated that pigs feed protected OAs supplementation has improved the growth performance [10,35,36], nutrient digestibility [11,35], and microflora counts [35].

## 4. The Application of Organic Acids in Swine

### 4.1. Organic Acid Usage in Swine to Improve Performance

It is reported that some of OAs are considered as a source of energy in pig gut because they are the intermediary products of tricarboxylic acid [42]. Kirchgessner (1982) noted that pigs can use fumaric acid as a source of energy as efficient as glucose [43]. Bosi et al. (1999) also indicated that the growth-promoting effects of the OAs were due to their energy values [44]. It is reported that it is possible that fumaric acid is an available energy source that may have positive impact on the mucosa in the small intestine, resulting in the absorptive surface and capacity improvement in the small intestine due to faster recovery of the gastrointestinal epithelial cells after weaning [45].

Previous researchers demonstrated positive influence of the dietary inclusion of OAs on the growth performance swine (Table 2). For instance, better results in growth performance, FI, and gain to feed ratio (G:F) in piglets fed the diet containing 0.4% OAs mixture (benzoic, citric, fumaric, lactic, and propionic acids) compared to the diet without OAs supplementation have been shown [46]. It is also demonstrated that the blend of OAs (including 10% malic, 13% citric, and 17% fumaric acids) has a positive influence on the growth performance on piglets (dietary 0.2 and 0.4% of OAs), growing pigs (dietary 0.2% of OAs), and finishing pigs (dietary 0.1 or 0.2% of OAs) [10,35,47]. Li et al. (2008) also indicated that weanling piglet fed the diet supplemented with 0.5% blends of OAs (butanic, fumaric, and benzioic acid) improved growth performance [48]. Feeding weaning pigs with the diet supplemented with an OAs blend (containing citric acid, calcium formate, and calcium lactate) and medium-chain fatty acids (MCFAs) (capric, lauric, and myristic acids) improved average daily gain (ADG), average daily feed intake (ADFI), and feed efficiency compared with those fed dietary zinc oxide addition [49]. It is reported that supplementation of 0.2% caprylic and 1.5% fumaric acids increased ADG compared to control diet in piglets [50].

In contrast, other studies observed there are no effects or even negative effects with individual OAs, such as citric, formic, or fumaric acids [51]. Zentek et al. (2013) also reported that in weanling piglets, there was no effect of 0.416% fumaric acid or 0.328% of lactic acid in the diet on growth performance [52]. In addition, it was reported that the growth performance of the weaning pigs that fed the diets containing a blend of fumaric, lactate, citric, propionic, and benzoic acids was not affected [46]. The inconsistent results might be partly due to differences in the ages of animals used, the types OAs used, and the doses of OAs and MCFAs used.

### 4.2. Organic Acids Usage in Swine to Improve Nutrient Digestibility

It has been mentioned that addition of OAs in the diet of pigs would have a positive effect on nutrient digestibility (Table 3). For instance, it is reported that supplementation with OAs (butyric, formic, and fumaric acids) enhanced apparent ileal digestibility of CP and essential amino acids up to 5% [57]. Dietary protected OA (containing 10% malic, 13% citric, and 17% fumaric acids) supplementation improved the digestibility of DM, N, and energy in finishing pigs at 12 weeks and lactating sows at weanling time [35,58], and the digestibility of CP, DM, fat, and energy in growing pigs [11]. Franco et al. (2005) indicated that supplementing the diets of weaning pigs with a blend of lactic acid, formic, and fumaric acids improved the digestibility of DM [59]. Likewise, it is reported that supplementation of OAs such as 2% benzoic acid and 0.5% in the diet of lactating sows and weanling pigs, improved nutrient digestibility [60,61].

In contrast, the other researchers reported that addition of OAs mixture (containing 10% malic, 13% citric, and 17% fumaric acids) to the diet of pigs and finishing pigs at 6 weeks of age had no effect on nutrient digestibility [35,36,47]. Kil et al also showed that weanling pigs fed the diet supplemented with OAs (containing: lactic, fumaric, and formic acids) did not affect the digestibility of DM and CP [62]. The different results could be due to the difference in diet composition, animals age, and animals state.

### 4.3. Effect of Organic Acids on Gut Microflora

Despite many years of research, the complex role of gastrointestinal microbial populations in nutrition and growth of the host animal is not completely understood. OAs could partially manage the nutrient competition by the gut microflora of animal (Table 4). For instance, it is reported that supplementation of 0.1% OAs mixture (10% malic, 13% citric, and 17% fumaric acids) to the diet of growing and finishing pigs decreased *E. coli* counts and increased Lactobacillus counts [36]. Upadhaya et al. (2014) found that dietary protected OAs supplementation (10% malic, 13% citric, and 17% fumaric acids) in finishing pigs diets reduced *E. coli* population [35]. Likewise, Ahmed et al. (2014) founded that weaned piglets fed the diet with 0.4% acidifier (including 4.1% propionic, 9.5% phosphoric, 10.2% lactic, and 17.2% formic acids) supplementation increased Bacilli and Lactobacilli concentrations and reduced Salmonella and *E. coli* counts [63]. It has been indicated that weaning pigs fed the diets containing 0.328% lactic and 0.416% fumaric acids had lower of colon *E. coli* [52]. Fecal *E. coli* counts reduction during the first phase and the improvement of fecal Lactobacillus counts in the second phase of the trial were also observed from pigs that were fed with the diet supplemented with protected OAs [47,55]. In addition, Devi et al. (2016) stated that protected OAs supplementation in sow’s diet decreased *E. coli* counts and increased Lactobacillus population in the farrowing and weaning periods [58].

### 4.4. Effect of Organic Acids on Fecal Gas Emission

There were also many studies noted that OAs supplementation reduced the environment issue by reducing noxious gas emission (Table 4). It is described that there is a significant reduction in noxious gases such as H_2_S when sows were fed the diets supplemented with protected OAs (10% malic, 13% citric, and 17% fumaric acids) at the end of farrowing [58]. Similarly, Upadhaya et al. (2014) noted that the protected OAs (10% malic, 13% citric, and 17% fumaric acids) administration in growing pigs’ diets reduced the emission of noxious gases [36]. Hossain et al. (2018) also indicated that growing pigs fed dietary protected OAs (10% malic, 13% citric, and 17% fumaric acids) supplementation resulted in the reduction of ammonia concentration [11]. The reduction of odorous NH_3_ and acetic gas emission was observed in finishing pigs fed the diet containing 0.2% OAs (10% malic, 13% citric, and 17% fumaric acids) [35]. Similarly, Eriksen et al. (2010) demonstrated that NH_3_ emission was reduced by 60–70% in the pigs fed the containing 2% benzoic acid [64]. The reduction in these odorous gases could possibly be due to a reduction in the population of the pathogenic bacterial in the gastrointestinal tract or due to enhancement of beneficial microbial activity and nutrient digestibility [35].

## 5. Conclusions

Different features of OAs have been investigated in the last decades. According to the literature, OAs have positive effects on reducing gastric pH, preventing the growth of pathogens, acting as an energy source during GIT intermediary metabolism, increasing apparent total tract digestibility (ATTD), and improving growth performance. However, the effect of OAs in practice is not always consistent due to the wide variety of available OAs products and the various recommended effective dosages with the different combinations. The efficacy of OAs could be influenced by type composition, dosage, formula, feeding regimen, environment, nutrient composition of feed, and the age and health status of animals. Besides, some studies have illustrated that use of microencapsulation technology to control the release of the core material to achieve the proper delay for the right stimulus, which could further enhance their effectiveness. Nevertheless, the available documents to date in feeding such compounds to swine seem to justify the assumption that OAs may have the potential to promote production performance and potential alternatives to replace antibiotics in the diets of animals.

## Figures and Tables

**Table 1:** List of acids and their properties.

Acid	Chemical Name	Formula	pKa
Tartaric	2,3-Dihydroxy-Butanedioic Acid	COOHCH(OH)CH(OH)COOH	2.93
Fumaric	2-Butenedioic Acid	COOHCH:CHCOOH	3.02
Citric	2-Hydroxy-1,2,3-Propanetricarboxylic Acid	COOHCH_2_C(OH)(COOH)CH_2_COOH	3.13
Malic	Hydroxybutanedioic Acid	COOHCH_2_CH(OH)COOH	3.40
Formic	Formic Acid	HCOOH	3.75
Lactic	2-Hydroxypropanoic Acid	CH_3_CH(OH)COOH	3.83
HMB	2-Hydroxy-4-Methylthio Butanoic Acid	CH_3_SCH_3_CH_2_CH(OH)COOH	3.86
Benzoic	Benzenecarboxylic acid	C_6_H_5_COOH	4.20
Acetic	Acetic Acid	CH_3_COOH	4.76
Sorbic	2,4-Hexandienoic Acid	CH_3_CH:CHCH:CHCOOH	4.76
Butyric	Butanoic Acid	CH_3_CH_2_CH_2_COOH	4.82
Propionic	2-Propanoic Acid	CH_3_CH_2_COOH	4.88

**Table 2:** Response of organic acid (OA) mixtures on growth performance and other parameters in pigs.

Composition of OAs Blends	Dose	Species	Growth Performance Improvements			Other Responses	Reference
			ADG	ADFI	G:F		
Fumaric, lactate, citric, propionic, and benzoic acids	0.2% 0.4%	Weaning pigs	Ns	Ns	Ns		Walsh et al [46]
Butanoic acid, fumaric acid, and benzoic acid,	0.5%	Weaning pigs	*	Ns	*		Li et al [48]
Caprylic acid and capric acid	0.1%	Weaning	*	-	Ns	Improved Villus in ileum	Hanczakowska et al [50]
Lactic acid, fumaric acid	1.05%	Weaning pigs	Ns	Ns	Ns	Increased fumaric acid in stomach and colon and decreased SCFA in colon. Improved *Bacteroides-Porphyromonas-Prevotella*, Clostridial cluster XIVa in stomach, Enterococci and Bifidobacteria in jejunum, Bacteroides-Porphyromonas-Prevotella in ileum, and Streptococci in colon.	Zentek et al [52]
Fumaric acid, citric acid, malic acid, capric acid, and caprylic acid	0.1% 0.2%	Growing pigs	*	Ns	Ns		Upadhaya et al [35]
Fumaric acid, citric acid, malic acid, capric acid, and caprylic acid	0.1%	Finishing pigs	*	Ns	*	Improved longissimus area and sensory evaluation of meat color	Upadhaya et al [36]
Fumaric acid, citric acid, malic acid, capric acid, and caprylic acid	0.1% 0.2% 0.3%	Growing pigs	Ns * Ns	Ns Ns Ns	Ns * Ns		Upadhaya et al [47]
Fumaric acid, citric acid, malic acid, capric acid, and caprylic acid	0.2% 0.4%	Weaning pigs	* *	* *	* *	Decreased diarrhea incidence	Lei et al [10]
Fumaric acid, citric acid, malic acid, capric acid, and caprylic acid	0.2%	Growing pigs	Ns	NS	*		Hossain et al [11]
Fumaric acid, citric acid, malic acid, capric acid, and caprylic acid	0.5% 1%	Finishing pigs	Ns	Ns	*		Lei et al [53]
Formic acid, acetic acid, propionic acid, and MCFA	0.3%	Weaning pigs	Ns	Ns	*	Increased acetic acid, propionic acid, isobutyric acid, butyric acid, total volatile fatty acid, total carbohydrates, acid detergent fiber, immunoglobulin, villus height/crypt depth. Reduced neutral detergent fiber, hydroxyl radicals	Long et al [54]
Fumaric acid, citric acid, malic acid, capric acid, and caprylic acid	0.1% 0.2%	Weaning pigs	Ns *	* *	Ns *		Upadhaya et al [55]
Benzoic acid, calcium formate, fumaric acid	0.15%	Weaning pigs	*	Ns	Ns	Increased calcium, phosphorus, ether extract digestibility, villus height	Xu et al [56]

ADG, average daily gain; ADFI, average daily feed intake, G:F, gain to feed ratio; Ns, no significance; *, *p* < 0.05; -, no measurement.

**Table 3:** Effects of OAs mixture on nutrient digestibility in pigs.

Composition of OAs Blend	Dose	Species	Nutrient Digestibility Response			References
			DM	N	E	
Caprylic acid and capric acid	0.1%	Weaning	Ns	-	-	Hanczakowska et al [50]
Fumaric acid, citric acid, malic acid, capric acid, and caprylic acid	0.1% 0.2%	Growing pigs	Ns	Ns	Ns	Upadhaya et al [36]
Fumaric acid, citric acid, malic acid, capric acid, and caprylic acid	0.1% 0.2%	Finishing pigs	*	*	*	Upadhaya et al [35]
Fumaric acid, citric acid, malic acid, capric acid, and caprylic acid	0.2%	Lactating sows	*	*	*	Devi et al [58]
Fumaric acid, citric acid, malic acid, capric acid, and caprylic acid	0.1% 0.2% 0.3%	Growing pigs	Ns	Ns	Ns	Upadhaya et al [47]
Fumaric acid, citric acid, malic acid, capric acid, and caprylic acid	0.2%	Growing pigs	*	*	*	Hossain et al [11]
Fumaric acid, citric acid, malic acid, capric acid, and caprylic acid	0.5% 1%	Finishing pigs	*	*	-	Lei et al [56]
Formic acid, acetic acid, propionic acid, and MCFA	0.3%	Weaning pigs	*	Ns	Ns	Long et al [54]
Fumaric acid, citric acid, malic acid, capric acid, and caprylic acid	0.2%	Weaning pigs	*	Ns	*	Upadhaya et al [55]

DM, dry matter; E, energy; N, nitrogen; Ns, no significance; *, *p* < 0.05; -, no measurement.

**Table 4:** Effects of OA mixtures on gut microflora and gas emission in pigs.

Composition of OAs Blend	Dose	Species	Gut Microflora and Gas Emission Response		Other Bacteria		References
Gut Microflora			*Lactobacillus*	*E. coli*			
Caprylic acid and capric acid	0.1%	Weaning Pigs	-	Ns	Decreased fungi in ileum		Hanczakowska et al [50]
Fumaric acid, citric acid, malic acid, capric acid, and caprylic acid	0.1% 0.2%	Finishing pigs	*	*			Upadhaya et al [35]
Fumaric acid, citric acid, malic acid, capric acid, and caprylic acid	0.2%	Lactating sows	*	*			Devi et al [58]
Fumaric acid, citric acid, malic acid, capric acid, and caprylic acid	0.1% 0.2% 0.3%	Growing pigs	*	Ns			Upadhaya et al [47]
Formic acid, acetic acid, propionic acid, and MCFAs	0.2% 0.3%	Weaning pigs	Ns	*	Increased total aerobic bacteria in feces		Long et al. [54]
Fumaric acid, citric acid, malic acid, capric acid, and caprylic acid	0.2%	Weaning pigs	*	*			Upadhaya et al [55]
**Fecal Gas Emission**			**NH_3_**	**H_2_S**	**R.SH**	**Acetic Acid**	
Fumaric acid, citric acid, malic acid, capric acid, and caprylic acid	0.1% 0.2%	Growing pigs	Ns	Ns	*	-	Upadhaya et al [36]
Fumaric acid, citric acid, malic acid, capric acid, and caprylic acid	0.1% 0.2%	Finishing pigs	*	-	-	*	Upadhaya et al [35]
Fumaric acid, citric acid, malic acid, capric acid, and caprylic acid	0.2%	Sows	*	*	Ns	Ns	Devi et al [58]
Fumaric acid, citric acid, malic acid, capric acid, and caprylic acid	0.2%	Growing pigs	*	Ns	-	*	Hossain et al [11]
Fumaric acid, citric acid, malic acid, capric acid, and caprylic acid	0.1% 0.2%	Weaning pigs	Ns	Ns	Ns	-	Upadhaya et al [55]

* *p* < 0.05; Ns, no significance; -, no measurement; R.SH, total mercaptans.
